# Inhibition of secreted phospholipase A2 by neuron survival and anti-inflammatory peptide CHEC-9

**DOI:** 10.1186/1742-2094-3-25

**Published:** 2006-09-11

**Authors:** Timothy J Cunningham, Jaquie Maciejewski, Lihua Yao

**Affiliations:** 1 Department of Neurobiology and Anatomy, Drexel University College of Medicine, 2900 Queen Lane, Philadelphia, PA 19129, USA

## Abstract

**Background:**

The nonapeptide CHEC-9 (CHEASAAQC), a putative inhibitor of secreted phospholipase A2 (sPLA2), has been shown previously to inhibit neuron death and aspects of the inflammatory response following systemic treatment of rats with cerebral cortex lesions. In this study, the properties of CHEC-9 inhibition of sPLA2 enzymes were investigated, using a venom-derived sPLA2 group I and the plasma of rats and humans as the sources of enzyme activity. The results highlight the advantages of inhibitors with uncompetitive properties for inflammatory disorders including those resulting in degeneration of neurons.

**Methods:**

Samples of enzyme and plasma were reacted with 1-Palmitoyl-2-Pyrenedecanoyl Phosphatidylcholine, a sPLA2 substrate that forms phospholipid vesicles in aqueous solutions. Some of the plasma samples were collected from restrained peptide-treated rats in order to confirm the validity of the *in vitro *assays for extrapolation to *in vivo *effects of the peptide. The enzyme reactions were analyzed in terms of well-studied relationships between the degree of inhibition and the concentrations of different reactants. We also examined interactions between different components of the reaction mixture on native polyacrylamide gels.

**Results:**

In all cases, the peptide showed the properties of an uncompetitive (or anti-competitive) enzyme inhibitor with Ki values less than 100 nanomolar. The electrophoresis experiments suggested CHEC-9 modifies the binding properties of the enzyme only in the presence of substrate, consistent with its classification as an uncompetitive inhibitor. Both the *in vitro *observations and the analysis of plasma samples from restrained rats injected with peptide suggest the efficacy of the peptide increases under conditions of high enzyme activity.

**Conclusion:**

Modeling studies by others have shown that uncompetitive inhibitors may be optimal for enzyme inhibition therapy because, unlike competitive inhibitors, they are not rendered ineffective by the accumulation of unmodified substrate. Such conditions likely apply to several instances of neuroinflammation where there are cascading increases in sPLA2s and their substrates, both systemically and in the CNS. Thus, the present results may explain the efficacy of CHEC-9 *in vivo*.

## Background

Pro-inflammatory enzymes and cytokines are increasingly attractive as therapeutic targets for a variety of inflammatory diseases and for the inflammatory component of neurodegenerative disorders. The 14–18 kD secreted phospholipase A2s (sPLA2s) are of interest in this regard because of their accessibility in the circulation and because local and systemic elevation of sPLA2s are associated with most forms of inflammation [1-5]. The secreted isoforms are part of a growing family of PLA2 enzymes whose activity leads to the production of several potent mediators of inflammation. Increased levels of extracellular sPLA2s have been detected in the plasma of patients affected by systemic inflammatory diseases such as acute pancreatitis, septic shock, extensive burns, and autoimmune diseases. The enzymes are also accumulated in inflammatory fluids such as the synovial fluid of patients with rheumatoid arthritis, the bronchoalveolar lavage of patients with bronchial asthma, and the nasal secretions of patients with allergic rhinitis. More recent experimental studies suggest sPLA2s are involved in traumatic and autoimmune precipitated neurodegeneration, and thus these enzymes are also a potential target for treatment of nervous system disorders.) [6,7,33].

CHEC-9 is a putative sPLA2 inhibitor that has recently been identified as an internal fragment of the survival-promoting, anti-inflammatory polypeptide DSEP/Dermcidin/PIF [6,8-12]. Following cerebral cortex lesions, a subcutaneous injection of CHEC-9 results in anti-inflammatory and neuron survival effects that last for at least 4 days, an effect due in part to an interruption of the inflammatory cascade that follows damage to the CNS. Given the efficacy of CHEC-9, the present study was undertaken to investigate CHEC-9 inhibition of sPLA2 activity in detail. The results suggest that CHEC-9 has several characteristics of an uncompetitive (or anti-competitive) sPLA2 inhibitor even when tested *ex vivo *with a chemically complex fluid like plasma. These properties are likely to be especially advantageous under conditions of inflammation and associated oxidative stress, and therefore are consistent with the peptide's performance *in vivo*.

## Methods

### Sources and preparation of sPLA2

Purified secreted phospholipase A2 group I from the venom of the Mozambique cobra (Naja mossambica) was obtained from Sigma. Blood was obtained from the trunk of 20 female Sprague Dawley rats (200–250 g) after decapitation, and by venipunture of 14 healthy adult humans of both sexes. Blood samples from 8 additional rats were collected following subcutaneous injections of 100 μg CHEC-9 or DMEM vehicle. These rats were placed in a standard rat restrainer during the collection period and the samples obtained via a tail nick. All specific procedures of this study were approved by both the Institutional Animal Care and Use Committee and by Institutional Review Board of Drexel University College of Medicine. Blood samples were treated with citrate-phosphate-dextrose anticoagulant (1:10, Sigma), and plasma prepared by centrifugation, before freezing at -80° until used in the enzyme assays. For the *ex vivo *studies, individual plasma samples were pooled from 3–7 rats or 3–5 humans.

### Enzyme assays

Enzyme assays were conducted at ambient temperature (22–25°) using a Victor 3 fluorescent reader (Perkin Elmer, Nutley NJ). The substrate was 1-Palmitoyl-2-Pyrenedecanoyl Phosphatidylcholine ("10-pyrene", Caymen Chemical, Ann Arbor MI) a substrate for all calcium dependent PLA2s with the exception of cPLA2 and PAF-AH. The substrate (supplied in chloroform) was dried under a nitrogen stream, quickly dissolved in ethanol, and stored at -20° prior to use. Substrate solutions were prepared in reaction buffer consisting of 50 mM tris (pH = 7.4), 0.1 M NaCl, 2 mM CaCl_2_, 0.25% fatty acid-free albumin (Sigma) and the CHEC-9 peptide at the indicated concentrations. CHEC-9 (CHEASAAQC) was synthesized by Celtek, (Nashville, TN), purified and cross-linked as described previously [6], and aliquots stored in tris buffer or DMEM vehicles at -80°. The 10-pyrene substrate forms phospholipids vesicles in aqueous solutions [13], and upon hydrolysis, releases 10-pyrenyldecanoic acid. This product is fluorescent in the presence of albumin and was measured at 350 nm excitation, 405 nM emission. Plasma samples were 20% final concentration in the reaction mixture, and all enzyme reactions were initiated with the addition of the substrate solution to the sPLA2 containing samples. Kinetic parameters including the properties of CHEC-9 were determined by measuring the initial velocities (Vo) of enzyme reactions (within 2 minutes of initiation). For experiments in which active sPLA2 enzyme concentration was measured in plasma samples from peptide-treated rats, we used a single substrate concentration and measured the steady-state rate of the enzyme reaction for 30 minutes. This rate is proportional to the concentration of active enzyme in the plasma if product formation during this period is linear with respect to time (see Fig. 6). In most experiments, relative fluorescent units (RFU) were converted to product concentration using a pyrenyldecanoic acid standard curve (Molecular Probes, Eugene, OR). For plasma, the background fluorescence of the plasma was not subtracted, but this did not effect the velocity measurements. Individual reactions were carried out in duplicate or triplicate and kinetic curves were produced using 5–6 substrate concentrations, with or without peptide, reacted simultaneously. Representative Lineweaver-Burke plots and nonlinear regression analyses of reactions using multiple peptide and substrate concentrations are presented in Results. Individual experiments were repeated 5 or more times with the same result, i.e., the direction of change of Km and Vmax was the same following inhibitor treatment, and Ki was less than 100 nM. Km and Vmax and r^2 ^were determined with regression software (Prism) from Graphpad (San Diego, CA).

### Identification of inhibitor properties

The characteristics of CHEC-9 inhibition were determined using both classical characterization of inhibitor types, i.e., the direction of changes in Km and Vmax, and more recent reports that derive consistent relationships between the extent of enzyme inhibition and substrate concentration [14-17]. The analyses of Geng [14] and Whitely[17] were particularly useful for the present studies because they allowed classification of CHEC-9 as well as calculation of the conventional inhibition constants for the various enzyme sources. We have repeated, combined, and rearranged some of their equations below in order to show the calculations used in the present experiments.

Ki_NR _is the apparent inhibition constant based on inhibition degree and is independent of inhibitor classification [14]. This value varies predictably with substrate concentration for different inhibitor types. It is defined as:

*K*i_NR _= [*I*]·*R*/(1-*R*), where [I] = inhibitor concentration, and

R = velocity_+inhibitor_/velocity_-inhibitor_.

This equation can be rewritten as:

*K*i_NR _= [*I*]·velocity_+inhibitor_/(velocity_-inhibitor _- velocity_+inhibitor_) for expressing Ki_NR _in terms of measured velocities. For the experiments in which the Michaelis constant Km was reliably estimated by nonlinear regression, Ki for an uncompetitive inhibitor was calculated from the following:

R = 1/(1 + ([I]/Ki·(1+(Km/[S])), where [S] = concentration of substrate.

This rearranges to: Ki = (R/(1-R))·[I]/(1+(Km/[S])), or, in terms of Ki_NR_:

Ki = Ki_NR_/(1+(Km/[S])).

### Polyacrylamide gel electrophoresis

Polyacrylamide gradient gels (5–15%) were run with and without SDS or reducing agents using sPLA2 group I alone or after mixing the enzyme with different combinations of the components of the reaction mixture (or their solvents). Samples prepared for the native gels were 50 μl containing 26 μM sPLA2, 40 μM CHEC-9, 560 μM 1-Palmitoyl-2-Pyrenedecanoyl Phosphatidylcholine (substrate), and 2 mM CaCl_2 _in 20 mM tris buffer (pH = 7.4), incubated together at room temperature for 30 min. After incubation, the samples were evaporated to 20 μl. The samples were then mixed with sample buffer containing only glycerol and bromophenol blue in 0.1 M tris (pH = 6.8) and loaded onto the gels. Following electrophoresis, the gels were washed, fixed, and stained with silver reagent according to conventional methods. The native gel experiment, using different reactant combinations run side by side (Fig. 3), was repeated four times with the same result.

## Results

### sPLA2 group I

**Figure 1:**
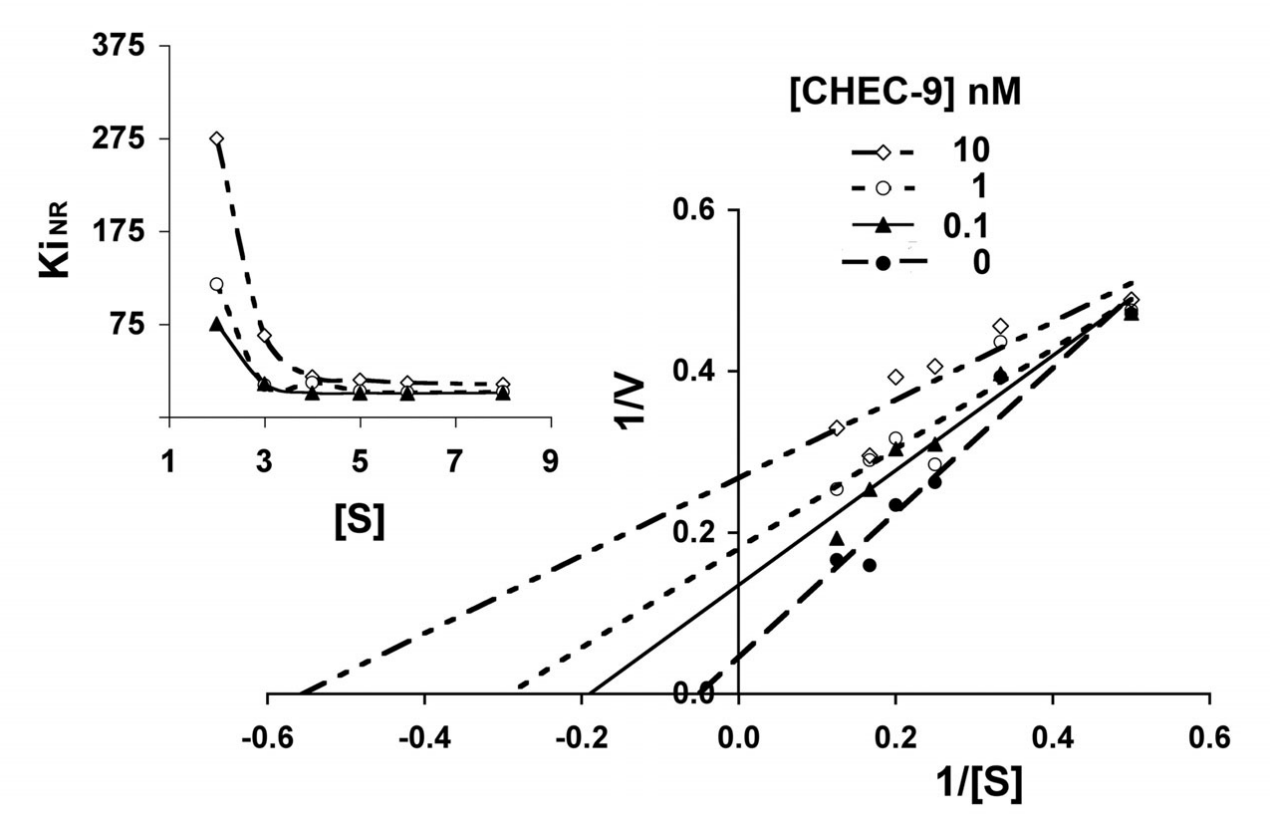
**Lineweaver-Burke double reciprocal plots showing CHEC-9 inhibition of sPLA2 group I**. The 10-pyrene substrate, at concentrations between 2 and 7 μM, was reacted with 5 nM sPLA2 and CHEC-9 at 0, 0.1, 1.0, and 10 nM. Plots of 1/Vo against 1/[substrate, S] gave convergent lines at higher values of 1/[S] (lower substrate concentrations) suggesting that the inhibition was dependent on substrate concentration. Plots of the apparent inhibition constant Ki_NR _vs. [S] (upper left) showed an overall decline in this value with increasing substrate concentration for each peptide concentration which is characteristic of an uncompetitive inhibitor. This is confirmed by computation of the kinetic parameters shown in Table 1 after fitting the data to the Michaelis-Menton hyperbola. [r^2^, p values for linearity of reciprocal plots: 0 nM – 0.945, 0.0012; 0.1 nM – 0.947, 0.0011; 1.0 nM – 0.868, 0.0069; 10 nM – 0.810, 0.0145].

The properties of CHEC-9 reacted with sPLA2 group I were examined using substrate concentrations that were one-half or less than the Michaelis constant (Km) measured during the course of the experiments, a relationship that is necessary for differentiating between different types of inhibitors [14]. Under these conditions, we found that the velocity of the enzyme reaction was reduced by nanomolar concentrations of CHEC-9 in the reaction mixture and the extent of inhibition depended on the concentration of both enzyme and substrate. For example, at the lowest substrate concentrations used in these experiments, CHEC-9 was minimally effective, ineffective, or even potentiated Vo. As a result, convergant lines appeared in the Lineweaver-Burke plots as 1/[S] increased (i.e., as substrate concentration decreased, Fig. 1). The intercepts of these plots suggested that CHEC-9 reduced both the Vmax and Km of the enzyme reaction, a result that was confirmed by nonlinear regression analyses after fitting all the data to the Michaelis-Menton equation (Table 1). This analysis suggested therefore that CHEC-9's properties were most consistent with an uncompetitive (or anti-competitive) inhibitor of sPLA2 group I. Furthermore, the apparent inhibition constant (Ki_NR_), calculated from the reaction velocities, varied inversely with the substrate concentration (Fig. 1, inset), another characteristic of an uncompetitive inhibitor[14].

**Table 1:** Kinetic Parameters at Different Inhibitor Concentrations

**Group I sPLA2**				
[CHEC-9] nM	**0.0**	**0.1**	**1.0**	**10.0**
Vmax nM/min/ng	21.67	11.54	5.56	4.04
Km μM	18.80	10.77	3.40	2.26
r^2^	0.896	0.942	0.851	0.752
				

Ki = 4.02 ± 1.56 nM				
				
**Human Plasma sPLA2**				

[CHEC-9] nM	**0.0**	**10.0**	**50.0**	

Vmax μM/min/ml	2.07	1.42	0.973	
Km	66.52	40.79	24.06	
r^2^	.981	.957	.970	

Ki = 62.01 ± 13.2 nM

r^2 ^= fit to Michaelis-Menton Equation

**Figure 2:**
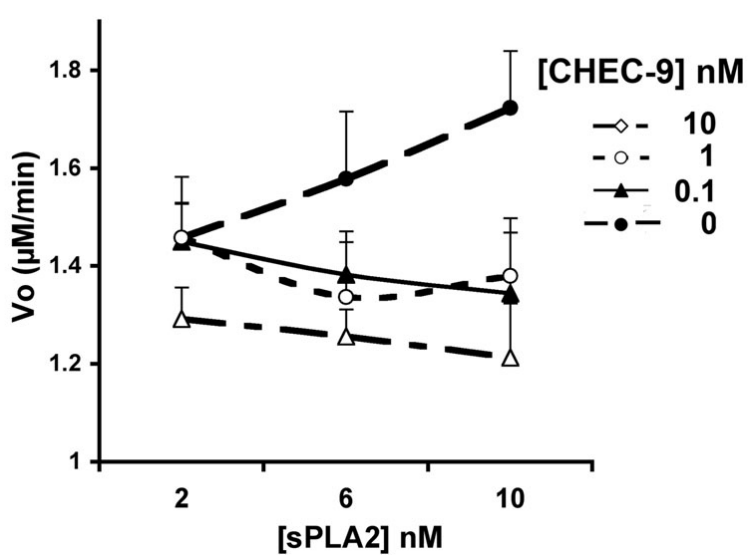
**Increasing efficacy of CHEC-9 with increasing enzyme concentrations**. The graph shows Vo for 3 different concentrations of enzyme reacted with 6 μM 10-Pyrene. Data points are mean ± s.e.m. of reactions with and without peptide (* indicates a significant difference, p < .05, t-test). All three concentrations of peptide were most effective at the highest concentration of sPLA2, and showed declining efficacy at the lower enzyme concentrations (see text).

Uncompetitive inhibitors are presumed to bind the enzyme-substrate complex so their efficacy is dependent on the levels of both enzyme and substrate in the reaction medium. In the next experiments, we determined effects of varying the enzyme concentration on CHEC-9 inhibition. We wanted to test the proposition that increasing the level of enzyme in the reaction, with a fixed substrate concentration, would make low concentrations of the inhibitor more effective due to increased availability of the enzyme-substrate complex. We measured velocities in reaction mixtures containing sPLA2 at 2, 6 and 10 nM mixed with CHEC-9 at 0, 0.1,1, and 10 nM (Fig. 2). For all concentrations of CHEC-9, maximal reductions of Vo occurred at 10 nM sPLA2, the highest concentration of enzyme tested. CHEC-9 concentrations lower than 10 nM, although minimally effective or ineffective at 2 nM or 6 nM sPLA2, produced consistent reductions in reaction velocity when used with 10 nM enzyme (Fig. 2). Thus increasing the enzyme concentration increased the efficacy of CHEC-9.

**Figure 3:**
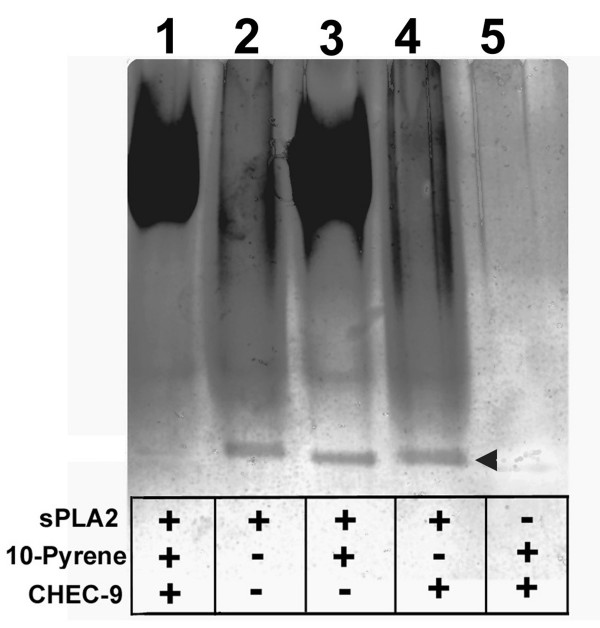
**Silver-stained SDS gel showing the migration of sPLA2 group I on native gels**. The enzyme was incubated with various components of the reaction mixture in calcium containing buffer as described in Methods. The sPLA2 band (arrowhead) disappeared or was markedly attenuated when the enzyme was pre-incubated with both peptide and substrate (lane 1), but not after incubation with buffer, substrate, or peptide alone (lanes 2, 3, 4). The gels suggested that the peptide could modify the structure and/or binding properties of the enzyme only in the presence of substrate.

### Polyacrylamide gel electrophoresis

In the electrophoresis experiments the concentrations of the participants in the reaction were scaled up so the migration of the enzyme on native gels could be observed following incubation with various reactants. We first confirmed that the sPLA2 used in these experiments was a single species after electrophoresis using conventional buffers with or without SDS and reducing reagents (not shown). The native gels had high argentophilic background but a single discrete sPLA2 band was still observed when the enzyme was pre-incubated in only the modified tris-calcium reaction buffer (without substrate or peptide, lane 2, Fig. 3). The same band appeared when either substrate or peptide alone was added to this mixture (lanes 3 and 4 arrowhead), although in the case of the former, a large, irregular, and intensely argentophilic band also appeared nearer the top of the gels, presumably representing a product or intermediate in the enzyme-substrate reaction. This same large band was also apparent when CHEC-9, enzyme and substrate were all present in the sample. However, the sPLA2 band was absent or at least dramatically reduced with CHEC-9 present (lane 1). Since the sPLA2 disappears or is diminished only in the presence of substrate and CHEC-9, it is suggested that peptide, substrate, and enzyme formed a complex that precluded the migration of the enzyme to its typical position in native gels. This result was consistent with the properties of an uncompetitive inhibitor.

### Human plasma

**Figure 4:**
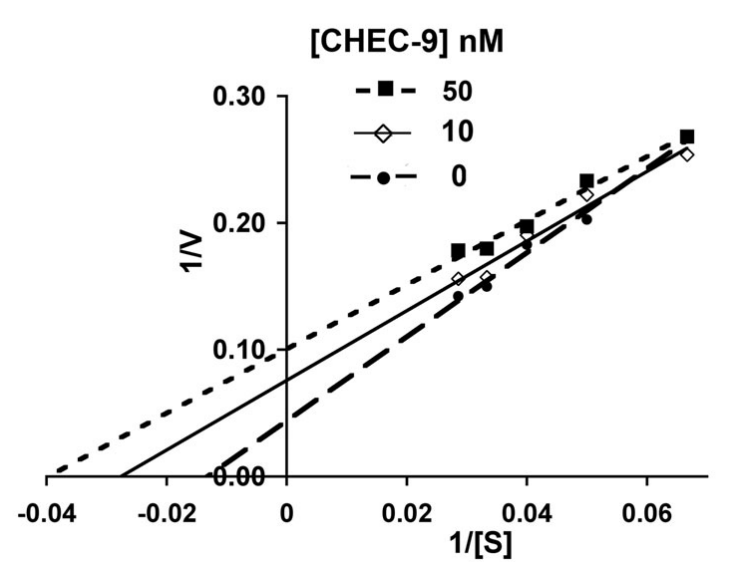
**Lineweaver-Burke double reciprocal plots showing CHEC-9 inhibition of sPLA2 activity in human plasma**. Plasma concentration was 20% of the reaction mixture, which also consisted of 10-pyrene at concentrations between 5–30 μM and CHEC-9 at 0, 10 and 50 nM. CHEC-9 was less effective or even exaggerated the reaction at the lower substrate concentrations causing the lines of the plot to converge with increasing values of 1/[S]. The kinetic parameters for reactions with human plasma are shown in Table 1 and are consistent with the properties of an uncompetitive inhibitor. [r^2^, p values for linearity of reciprocal plots: 0 nM – 0.987, 0.006; 10 nM – 0.967, 0.0026; 50 nM – 0.980, 0.0012].

Human plasma required higher concentrations of substrate while reaching lower reaction velocities than sPLA2 group I. The Km values calculated for these plasma reactions were therefore higher. However, the pattern of inhibition of enzyme activity was nearly identical to that obtained with purified sPLA2 (Fig. 4). In the first place, the peptide produced exaggerated enzyme activity in human plasma at the lowest substrate concentrations used. This is a classical feature of uncompetitive inhibition because at low substrate concentrations the mass action effects of the inhibitor facilitated formation of the ES complex, which elevated the initial reaction velocity (Vo) compared to the reaction without peptide. Most importantly, Km and Vmax of plasma samples were both reduced by CHEC-9 under these conditions (Table 1), which is the hallmark of the uncompetitive inhibitor.

### Rat plasma

**Figure 5:**
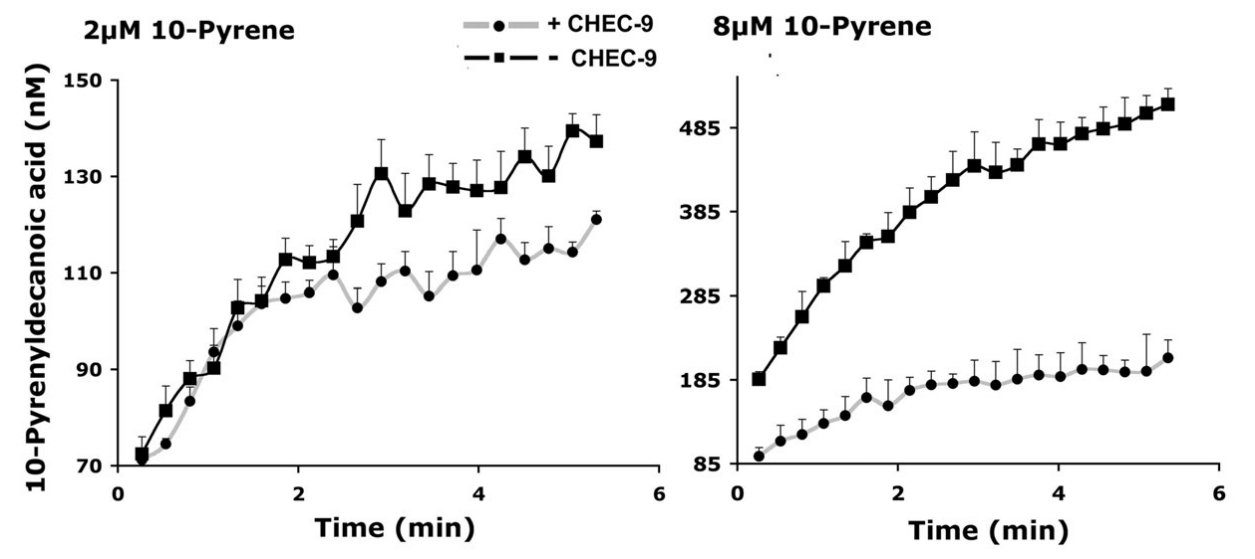
**Enzyme reactions using rat plasma with and without 25 nM CHEC-9 added to the reaction mixture**. These reactions were characterized by delayed inhibition at the lower substrate concentrations, which is consistent with uncompetitive inhibition (see text). Each point represents the mean ± s.e.m. of three reactions.

The kinetic behaviour of rat plasma reacted with the 10-pyrene substrate was complex. The expected progression of activity with increasing substrate concentration was often interrupted abruptly at high substrate concentrations (> 25 μM) by profound inhibition of activity, even without peptide treatment. CHEC-9 would sometimes reverse this inhibitory effect, suggesting a complex competitive relationship between substrate, rat plasma sPLA2(s), and CHEC-9 under these conditions. Nonetheless, during individual reactions at lower substrate concentrations, when product formation was relatively stable, inhibition by CHEC-9 was readily observed. The characteristics of these individual enzyme reactions were consistent with the properties of CHEC-9 described above. For example, at very low substrate concentrations, CHEC-9 inhibition of rat plasma sPLA2 was delayed (Fig. 5, left panel). The delay may indicate the requirement for formation of a sufficient level of the ES complex, as also suggested in the experiments above. With increased substrate concentrations (right panel) this delay of the inhibition is not detected, as quantities of the ES complex necessary for inhibition by the peptide are present instantaneously (within the present limits of detection). This situation is in fact equivalent to the inhibition of Vo in the previous experiments, which was most apparent at higher substrate concentrations.

**Figure 6:**
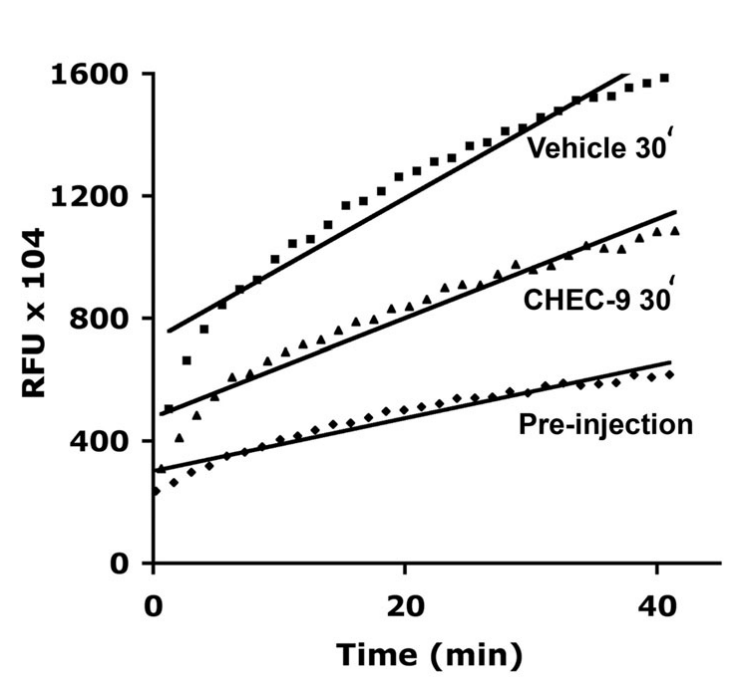
**Linear formation of reaction product over time in plasma samples from rats injected with CHEC-9 or vehicle**. The rats were restrained for blood sampling and then injected with 100 μg peptide or equivalent volume of vehicle under the skin of the back. The reactions of plasma showed a roughly linear increase in product for the period of measurement using 15 μM of the 10-pyrene substrate. Therefore the concentration of active enzyme in the plasma is proportional to the rate of the reaction. Examples shown are samples from peptide and vehicle-treated rats taken 30 minutes after treatment, and a plasma sample from a vehicle injected animal prior to treatment. RFU = relative fluorescent units.

### *In vivo *experiments

**Figure 7:**
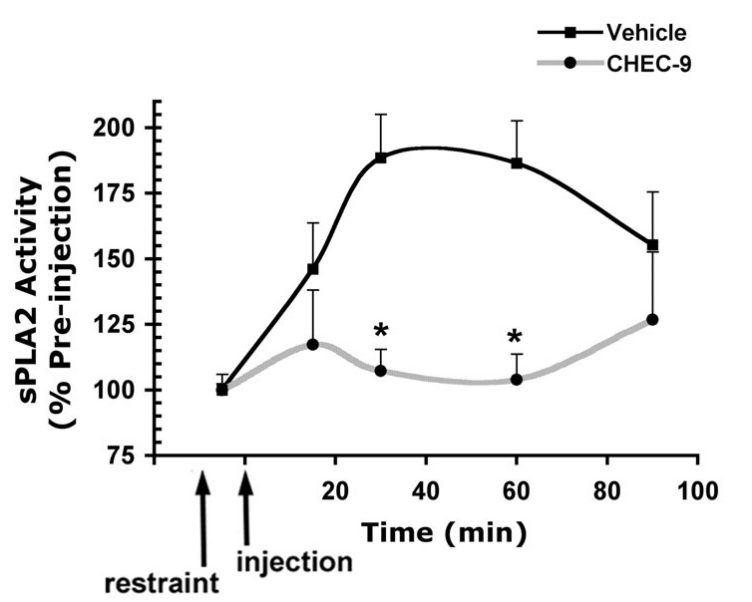
**Reduced levels of active sPLA2 enzyme in rat plasma samples after systemic delivery of CHEC-9**. A transient rise in mean levels of active enzyme was found in the first 60 min after vehicle injections in restrained rats. CHEC-9 inhibits this increase at 30 and 60 min. Each data point represents the mean ± s.e.m. of 4 rats (2–3 measurements/time pt./rat) in each of the two treatment groups. The values are expressed as a percent of the pre-injection samples for individual rats. (*) represents significant differences at 30 and 60 minutes (p < 0.05 by Mann Whitney test).

In order to verify that the results of these *in vitro *and *ex vivo *assay procedures were appropriate for comparison to the *in vivo *effects of CHEC-9, we injected rats with peptide (100 μg) or vehicle, collected plasma samples, and then used the 10-pyrene substrate to measure sPLA2 activity. For these samples, the rate of product formation was determined over a 30-minute period in the presence of 15 μM substrate. We confirmed that the reaction was linear during the period of measurement so the measured rate of the reaction was proportional to the concentration of active enzyme in the plasma (Fig. 6). Samples collected at 15–30 min intervals following treatment showed a transient elevation in sPLA2 levels at 30 and 60 minutes, presumably due to the stress of restraint, blood sampling, and/or the injection procedures (Fig. 7). This increase was inhibited in the peptide-injected rats. In these rats, CHEC-9 appeared to 'buffer' the sPLA2 activity, holding the level of active enzyme, on average, near pre-injection values. These results are also consistent with the activity-dependent inhibition by the peptide, as suggested by the *in vitro *and *ex vivo *experiments outlined above.

## Discussion

### Competitive versus uncompetitive inhibitors

Studies of enzyme inhibitors in open systems have suggested that competitive inhibitors, still a major focus of therapeutic drug design, have inherent limitations that may compromise their efficacy [18]. These limitations involve the accumulation of unmodified substrate, a natural result of blocking of the enzyme binding to substrate by the inhibitor. The accumulated substrate will eventually compete successfully for the enzyme overcoming the inhibition. These limitations are expected to be especially apparent in situations of both acute and persistent inflammation, the conditions that are amenable to sPLA2 inhibition therapy. Even a more or less localized inflammatory lesion is accompanied by both a localized and systemic sPLA2 response as has been demonstrated for a variety of disorders, including following nervous system lesions (see Introduction). Inflammation and associated oxidative stress will also increase levels of sPLA2 substrates including, (but not exclusively), acidic phospholipids expressed at the cell surface [19], phosphatidic acid released by activated immune cells[20,21], and oxidized low density lipoproteins, components of the phospholipid targets in circulating lipoproteins [22,23]. Successful enzyme inhibition therapy, introduced systemically, must therefore overcome widespread elevations in enzyme and available substrates. The uncompetitive sPLA2 inhibitor may have an advantage under such conditions, since this increased activity may actually favor the enzyme inhibition. Therefore, competitive sPLA2 inhibitors, even those displaying potent inhibition *in vitro*, may have short-lived effects *in vivo *depending on the level and persistence of the inflammatory response. It is possible that this limitation contributed to the lack of *in vivo *efficacy of a number of potent small molecule competitive sPLA2 inhibitors developed commercially and abandoned [4], as well as to the poor performance of competitive sPLA2 inhibitors in recent clinical trials [4,24,25]. It should be noted however that these inhibitors were not tested in models of neurodegenerative disease.

### Neurodegeneration and PLA2 isoforms

The survival-promoting and anti-inflammatory effects of CHEC-9 are most readily explained by the inhibition of PLA2 activity, either sPLA2s directly, or cytosolic PLA2 which may be regulated by sPLA2s during oxidative stress [19,26,27]. The activity of the PLA2 enzymes has been associated with cell degeneration in many systems including the nervous system [28,29]. Furthermore, microglia and macrophages may depend on PLA2 activity for cell killing [30,31]. At present, the PLA2 isoforms targeted by CHEC-9, where *in vivo *they are targeted, and their relevance to particular neurodegenerative disorders is unknown. We have emphasized *ex vivo *experiments with plasma and sPLA2 activity in plasma after *in vivo *exposure to CHEC-9 because these fluids, while complex in terms of the number of active PLA2 isoforms present [2,22], likely contribute to the systemic component of neuroinflammatory disorders. In fact, the efficacy of CHEC-9 in plasma, as well as with a venom-derived sPLA2, indicates broad specificity of the peptide, which may also contribute to its effectiveness *in vivo*. The nonenzymatic functions of PLA2 enzymes may also contribute to the pathophysiology of neurodegenerative diseases, and CHEC-9 could also influence these activities at the same time or independently of enzyme inhibition [2,32].

## Conclusion

The contribution of sPLA2 enzyme activity to inflammatory and degenerative disorders of the nervous system is increasingly appreciated. Given the nature of inflammatory stimuli and of the inflammatory cascade, inhibitors of enzyme activity with uncompetitive properties may be optimal for therapeutic intervention, since their efficacy is increased under conditions of escalating enzyme activity.

## Competing interests

TJC, LY, and Drexel University have applied for patent protection of CHEC-9, the peptide inhibitor used in these experiments.

## Authors' contributions

JM conducted the enzyme assays and LY was responsible for the in vivo studies. TJC organized the data following computer analysis and wrote the manuscript. All authors contributed to design of the experiments and interpretation of data, and trouble-shooting the specific procedures involved. All authors approved of the final version of the manuscript.

## References

[B1] Corke C, Glenister K, Watson T (2001). Circulating secretory phospholipase a(2) in critical illness – the importance of the intestine. Crit Care Resusc.

[B2] Triggiani M, Granata F, Giannattasio G, Marone G (2005). Secretory phospholipases A2 in inflammatory and allergic diseases: not just enzymes. J Allergy Clin Immunol.

[B3] Touqui L, Alaoui-El-Azher M (2001). Mammalian secreted phospholipases A2 and their pathophysiological significance in inflammatory diseases. Curr Mol Med.

[B4] Springer DM (2001). An update on inhibitors of human 14 kDa Type II s-PLA2 in development. Curr Pharm Des.

[B5] Meyer MC, Rastogi P, Beckett CS, McHowat J (2005). Phospholipase A2 inhibitors as potential anti-inflammatory agents. Curr Pharm Des.

[B6] Cunningham TJ, Souayah N, Jameson B, Mitchell J, Yao L (2004). Systemic treatment of cerebral cortex lesions in rats with a new secreted phospholipase A2 inhibitor. J Neurotrauma.

[B7] Pinto F, Brenner T, Dan P, Krimsky M, Yedgar S (2003). Extracellular phospholipase A2 inhibitors suppress central nervous system inflammation. Glia.

[B8] Cunningham TJ, Hodge L, Speicher D, Reim D, Tyler-Polsz C, Levitt P, Eagleson K, Kennedy S, Wang Y (1998). Identification of a survival-promoting peptide in medium conditioned by oxidatively stressed cell lines of nervous system origin. J Neurosci.

[B9] Cunningham TJ, Jing H, Akerblom I, Morgan R, Fisher TS, Neveu M (2002). Identification of the human cDNA for new survival/evasion peptide (DSEP): studies in vitro and in vivo of overexpression by neural cells. Exp Neurol.

[B10] Landgraf P, Sieg F, Wahle P, Meyer G, Kreutz MR, Pape HC (2005). A maternal blood-borne factor promotes survival of the developing thalamus. Faseb J.

[B11] Schittek B, Hipfel R, Sauer B, Bauer J, Kalbacher H, Stevanovic S, Schirle M, Schroeder K, Blin N, Meier F (2001). Dermcidin: a novel human antibiotic peptide secreted by sweat glands. Nat Immunol.

[B12] Lowrie AG, Wigmore SJ, Wright DJ, Waddell ID, Ross JA (2006). Dermcidin expression in hepatic cells improves survival without N-glycosylation, but requires asparagine residues. Br J Cancer.

[B13] Radvanyi F, Jordan L, Russo-Marie F, Bon C (1989). A sensitive and continuous fluorometric assay for phospholipase A2 using pyrene-labeled phospholipids in the presence of serum albumin. Anal Biochem.

[B14] Geng W (2003). A method for identification of inhibition mechanism and estimation of Ki in in vitro enzyme inhibition study. Drug Metab Dispos.

[B15] Cortes A, Cascante M, Cardenas ML, Cornish-Bowden A (2001). Relationships between inhibition constants, inhibitor concentrations for 50% inhibition and types of inhibition: new ways of analysing data. Biochem J.

[B16] Ito K, Iwatsubo T, Kanamitsu S, Ueda K, Suzuki H, Sugiyama Y (1998). Prediction of pharmacokinetic alterations caused by drug-drug interactions: metabolic interaction in the liver. Pharmacol Rev.

[B17] Whiteley CG (2000). Enzyme kinetics: partial and complete uncompetitive inhibition. Biochem Educ.

[B18] Westley AM, Westley J (1996). Enzyme inhibition in open systems. Superiority of uncompetitive agents. J Biol Chem.

[B19] Kuwata H, Sawada H, Murakami M, Kudo I (1999). Role of type IIA secretory phospholipase A2 in arachidonic acid metabolism. Adv Exp Med Biol.

[B20] Kusner DJ, Hall CF, Jackson S (1999). Fc gamma receptor-mediated activation of phospholipase D regulates macrophage phagocytosis of IgG-opsonized particles. J Immunol.

[B21] Snitko Y, Yoon ET, Cho W (1997). High specificity of human secretory class II phospholipase A2 for phosphatidic acid. Biochem J.

[B22] Pruzanski W, Lambeau L, Lazdunsky M, Cho W, Kopilov J, Kuksis A (2005). Differential hydrolysis of molecular species of lipoprotein phosphatidylcholine by groups IIA, V and X secretory phospholipases A2. Biochim Biophys Acta.

[B23] Memon RA, Staprans I, Noor M, Holleran WM, Uchida Y, Moser AH, Feingold KR, Grunfeld C (2000). Infection and inflammation induce LDL oxidation in vivo. Arterioscler Thromb Vasc Biol.

[B24] Zeiher BG, Steingrub J, Laterre PF, Dmitrienko A, Fukiishi Y, Abraham E (2005). LY315920NA/S-5920 a selective inhibitor of group IIA secretory phospholipase A2, fails to improve clinical outcome for patients with severe sepsis. Crit Care Med.

[B25] Bradley JD, Dmitrienko AA, Kivitz AJ, Gluck OS, Weaver AL, Wiesenhutter C, Myers SL, Sides GD (2005). A randomized, double-blinded, placebo-controlled clinical trial of LY333013 a selective inhibitor of group II secretory phospholipase A2, in the treatment of rheumatoid arthritis. J Rheumatol.

[B26] Fonteh AN, Atsumi G, LaPorte T, Chilton FH (2000). Secretory phospholipase A2 receptor-mediated activation of cytosolic phospholipase A2 in murine bone marrow-derived mast cells. J Immunol.

[B27] Han WK, Sapirstein A, Hung CC, Alessandrini A, Bonventre JV (2003). Cross-talk between cytosolic phospholipase A2 alpha (cPLA2 alpha) and secretory phospholipase A2 (sPLA2) in hydrogen peroxide-induced arachidonic acid release in murine mesangial cells: sPLA2 regulates cPLA2 alpha activity that is responsible for arachidonic acid release. J Biol Chem.

[B28] Cummings BS, McHowat J, Schnellmann RG (2000). Phospholipase A(2)s in cell injury and death. J Pharmacol Exp Ther.

[B29] Bazan NG, Colangelo V, Lukiw WJ (2002). Prostaglandins and other lipid mediators in Alzheimer's disease. Prostaglandins Other Lipid Mediat.

[B30] Klegeris A, McGeer PL (2000). Interaction of various intracellular signaling mechanisms involved in mononuclear phagocyte toxicity toward neuronal cells. J Leukoc Biol.

[B31] Klegeris A, McGeer PL (2003). Toxicity of human monocytic THP-1 cells and microglia toward SH-SY5Y neuroblastoma cells is reduced by inhibitors of 5-lipoxygenase and its activating protein FLAP. J Leukoc Biol.

[B32] Fuentes L, Hernandez M, Nieto ML, Sanchez Crespo M (2002). Biological effects of group IIA secreted phosholipase A(2). FEBS Lett.

[B33] Cunningham TJ, Yao L, Oetinger M, Cort L, Blankenhorn EP, Greenstein JI (2006). Secreted phospholipase A2 activity in experimental autoimmune encephalomyelitis and multiple sclerosis. J Neuroinflammation.

